# Supplementary material for: Directed evolution of and structural insights into antibody-mediated disruption of a stable receptor-ligand complex
Source: Nat Commun. 2021 Dec 3;12:7069. doi: 10.1038/s41467-021-27397-z (PMC8642555; doi:10.1038/s41467-021-27397-z)
Supplement: Supplementary file 1 — Supplementary Information [file 41467_2021_27397_MOESM1_ESM.pdf]

Supplementary Information

Directed evolution of and structural insights into antibody-mediated disruption of a stable receptor-ligand complex

Authors: Luke F. Pennington[1,2,3], Pascal Gasser[4,5], Silke Kleinboelting[1], Chensong Zhang[6], Georgios Skiniotis[6], Alexander Eggel[4,5] & Theodore S. Jardetzky[1,2,3*]

Affiliations:
[1]Department of Structural Biology, Stanford University School of Medicine, Stanford California 94305.
[2]Progam in Immunology, Stanford University School of Medicine, Stanford California 94305.
[3]Sean N. Parker Center for Allergy Research at Stanford University.
[4]Department of Rheumatology, Immunology and Allergology, University Hospital Bern, Switzerland.
[5]Department of BioMedical Research, University of Bern, Switzerland.
[6]Department of Molecular and Cellular Physiology, Stanford University School of Medicine, Stanford California 94305.
*Correspondence to: TSJ - Department of Structural Biology, Stanford University School of Medicine, Stanford, CA 94305; Telephone: 650-498-4179; Fax: 650-723-4943; e-mail: tjardetz@stanford.edu

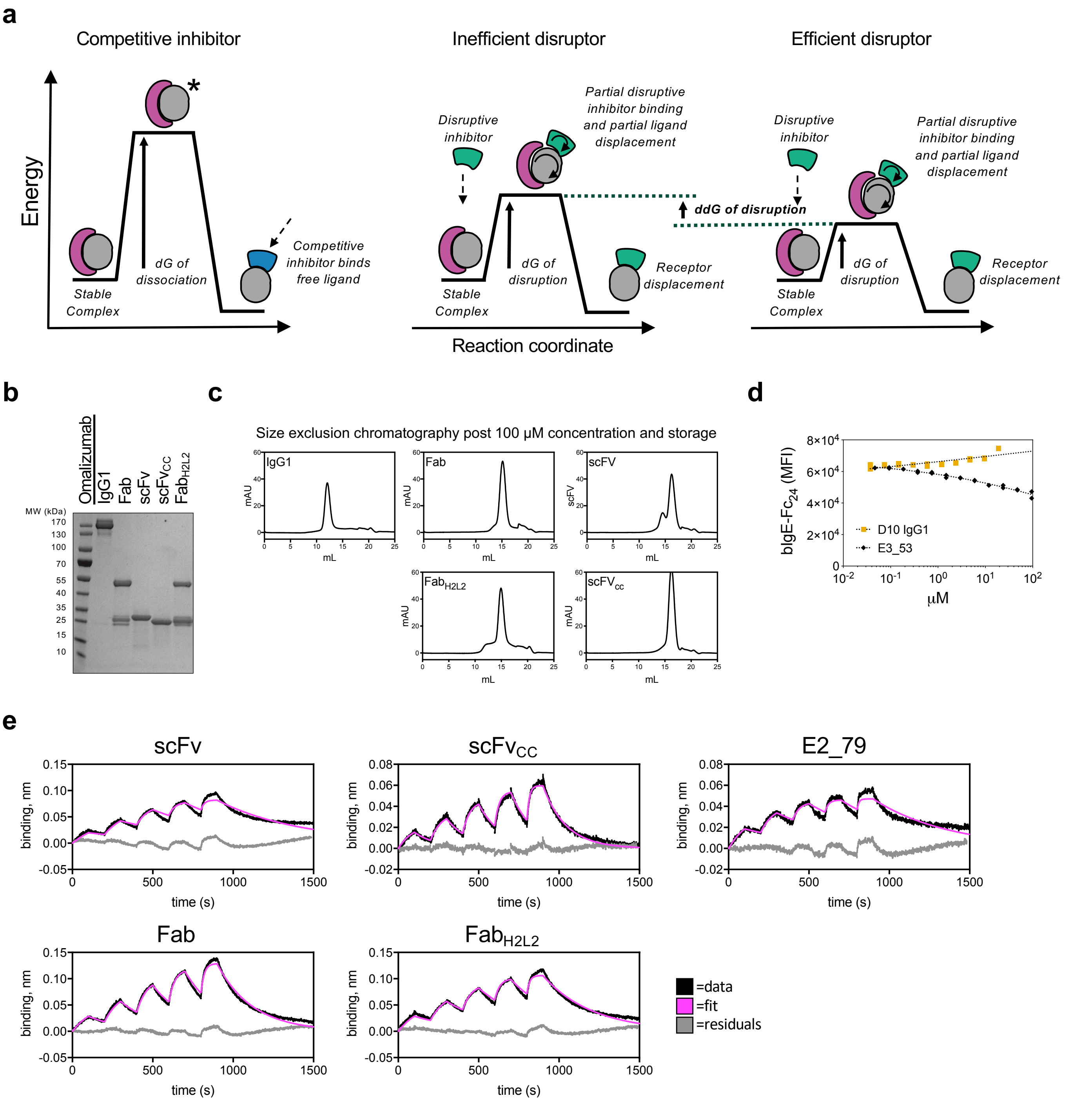

Supplementary Figure 1. Schematic of inhibition vs. disruption and the analysis of soluble recombinant omalizumab variants. a (left) Schematic free energy diagram of a competitive inhibitor, which requires the dissociation of a stable ligand receptor complex prior to binding. (right) Energy diagram of facilitated dissociation by an inefficient disruptor and an efficient disruptor. Here dG of disruption represents the free energy barrier imposed on a disruptive inhibitor by the steric overlap with the receptor and ddG reflects the difference in this energy barrier between two disruptors of different efficiencies. **b** Non-reducing SDS-PAGE gel of size exclusion chromatography (SEC) purified species, note partial dissociation of Fab species lacking hinge disulfide into VH/VL despite monomer peak by SEC. **c** Size exclusion chromatography (Superdex S200 10/300 GL) from all variants after concentration to 100 µM for disruption assays. **d** Disruption curves showing loss of bIgE-$Fc_{2\text{-}4}$ bound to FcεRIα-Ova coated polystyrene beads after 30-minute treatment with indicated controls. Curve fits (dotted line) from replicate assays. **e** Biotinylated full length recombinant IgE was immobilized on streptavidin (SA) tips and single cycle kinetic analysis (SCK) binding experiments were conducted on the Octet Red 96, exported, and fit in BiaEvaluation 3.0. Each SCK experiment contained five 100 s association cycles, four 100 s dissociation cycles, and a final 600 s dissociation cycle and each experiment was conducted with two-fold serial dilutions from 50 nM to 1.56 nM, binding data was reference subtracted to parallel blank tips exposed to same serial analyte dilutions. Source data are provided as a Source Data file.

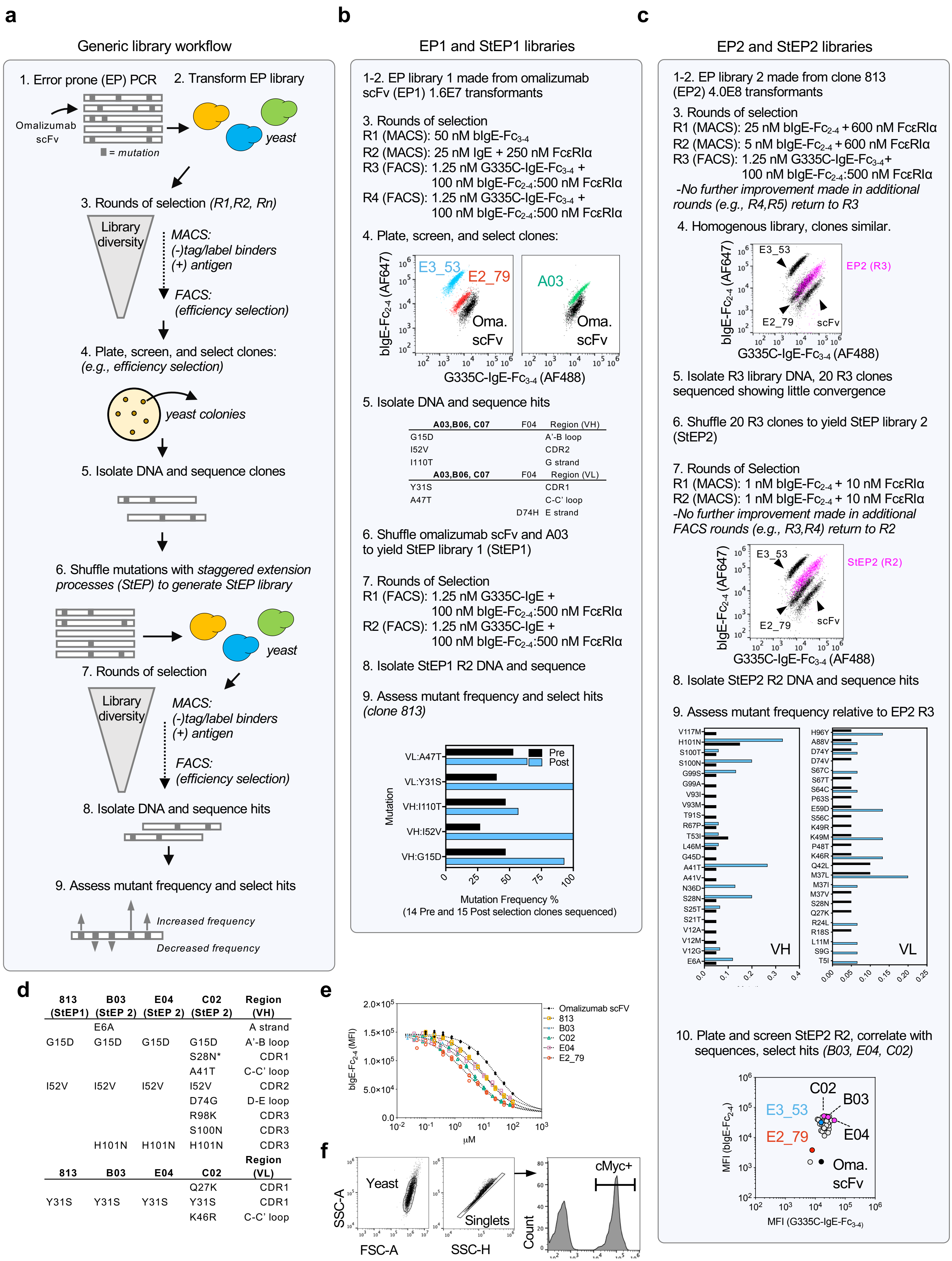

Supplementary Figure 2. (Legend on next page)

Supplementary Figure 2. Directed evolution of omalizumab variants. a Schematic of library construction, selection, and sequencing with steps 1-9 numbered. **b** Overview of error-prone-1 (EP1) and staggered-extension-process-1 (StEP1) library construction, selection, and sequencing. **(b4)** Clones from R4 of EP1 library and controls stained with the disruption efficiency stain [G335C-IgE-Fc_{3-4} (1.25 nM) and bIgE-Fc_{2-4}:FcεRIα-Ova (100 nM: 500 nM)](left). One of the R4 hits (clone A03) is depicted compared to omalizumab scFv(right). **(b5)** VH and VL mutations of selected clones from **(b4)** relative to omalizumab scFv. **(b9)** Pre and post selection StEP1library mutation frequencies. **c** Overview of error-prone-2 (EP2) and staggered-extension-process-2 (StEP2) library construction, selection, and sequencing. **(c4)** R3 of EP2 and controls stained with disruption efficiency stain [G335C-IgE-Fc_{3-4} (1.25 nM) and bIgE-Fc_{2-4}:FcεRIα-Ova (100 nM: 500 nM)]. **(c7)** R2 of StEP2 library stained as in (C4). **(c9)** Relative mutation frequencies from EP2 (R2) and StEP2 (R2) clones. **(c10)** MFI (for clarity) of clones from R2 of the StEP2 library plotted versus controls, with selected hits highlighted (magenta). **d** VH and VL mutations of clones selected in (B and C) **e** Disruption curves showing loss of bIgE-Fc2-4 bound to FcεRIα-Ova coated polystyrene beads after 30-minute treatment with anti-IgE. Curve fits (dotted line). **f** Gating scheme for yeast flow cytometry plots.

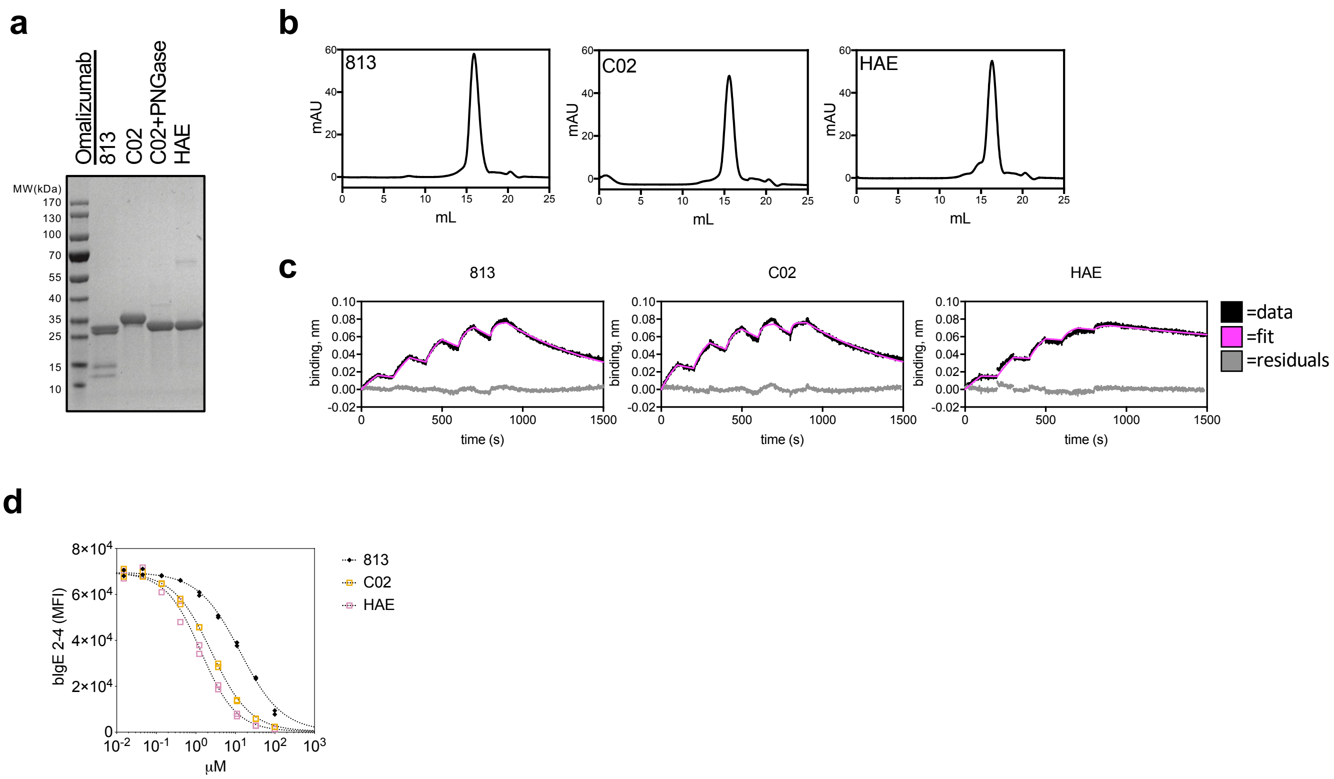

Supplementary Figure 3. Expression and validation of omalizumab library hits and HAE.
a Non-reducing SDS-PAGE gel of scFv variants. **b** Size exclusion chromatography (Superdex S200 10/300 GL or HiLoad 16/600 when noted) for all variants after concentration to 100 μM for disruption assays. **c** Biotinylated full length recombinant IgE was immobilized on SA-tips and single cycle kinetic analysis (SCK) binding experiments were conducted on the Octet Red 96, exported, and fit in BiaEvaluation 3.0. Each SCK experiment contained five 100s association cycles, four 100s dissociation cycles, and a final 600s dissociation cycle. Experiments were conducted with two-fold serial dilutions from 50 nM to 1.56 nM, and binding data was reference subtracted to parallel blank tips exposed to same serial analyte dilutions. **d** Disruption curves showing loss of bIgE-Fc_{2-4} bound to FcεRIα-Ova coated polystyrene beads after 30-minute treatment with omalizumab variants. Curve fits (dotted line) from replicate assays. Source data are provided as a Source Data file.

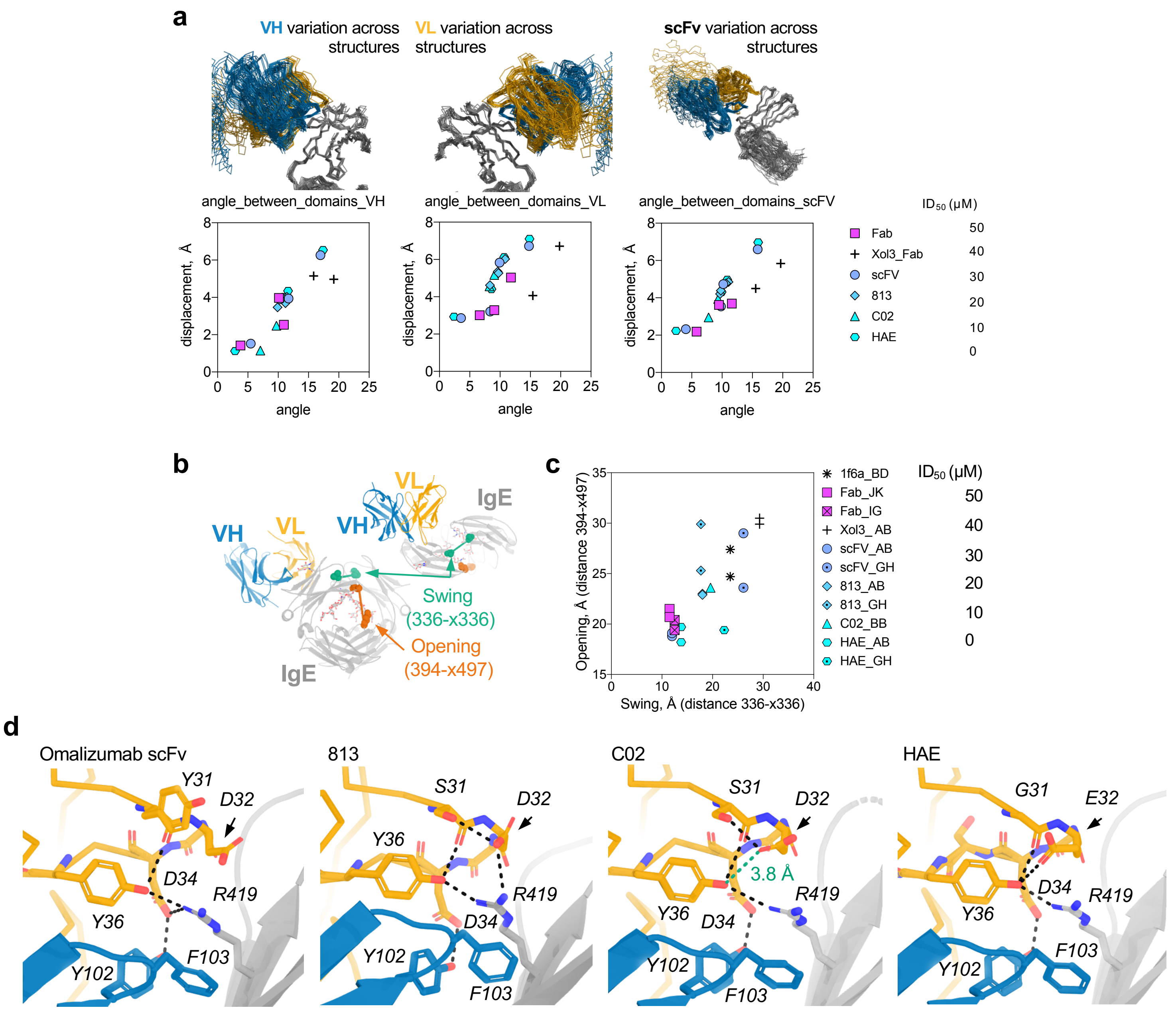

Supplementary Figure 4. Conformational rearrangements in high affinity disruptive omalizumab variant structures. a Ribbon diagram of all omalizumab:IgE pairs aligned at Cε3 highlighting relative positions of VH, VL, or scFvs across structures, with the relative displacement of domains measured as compared to omalizumab:Fab complex (5HYS) chains H (VH), L (VL), or H+L (ScFv) using *angle_between_domains* from Pymol ScrIpt Collection. The resulting angles and displacements were plotted and color-mapped by the mean ID_{50} of each variant in bead-based disruption assays. **b** Schematic of measured "swing," and "opening," distances on scFv:IgE-$Fc_{3\text{-}4}$ structures. **c** The swing and opening distances of the IgE-Fc in antibody and receptor co-complexes was plotted and color mapped by the disruptive potency of each antibody when possible, with chain pairs indicated. **d** Details of hydrogen bonding network around the R419 binding pocket across omalizumab scFv and scFv variants. A shared Y31D/G mutation enables repositioning of residue D/E 32 in all variants and facilitates the formation of intrachain polar bonds to adjacent residues within neighboring CDR1 loop. Black dashes represent predicted polar bonds, and green dashes represent a potential polar bond falling beyond the 3.5 Å cutoff. Source data are provided as a Source Data file.

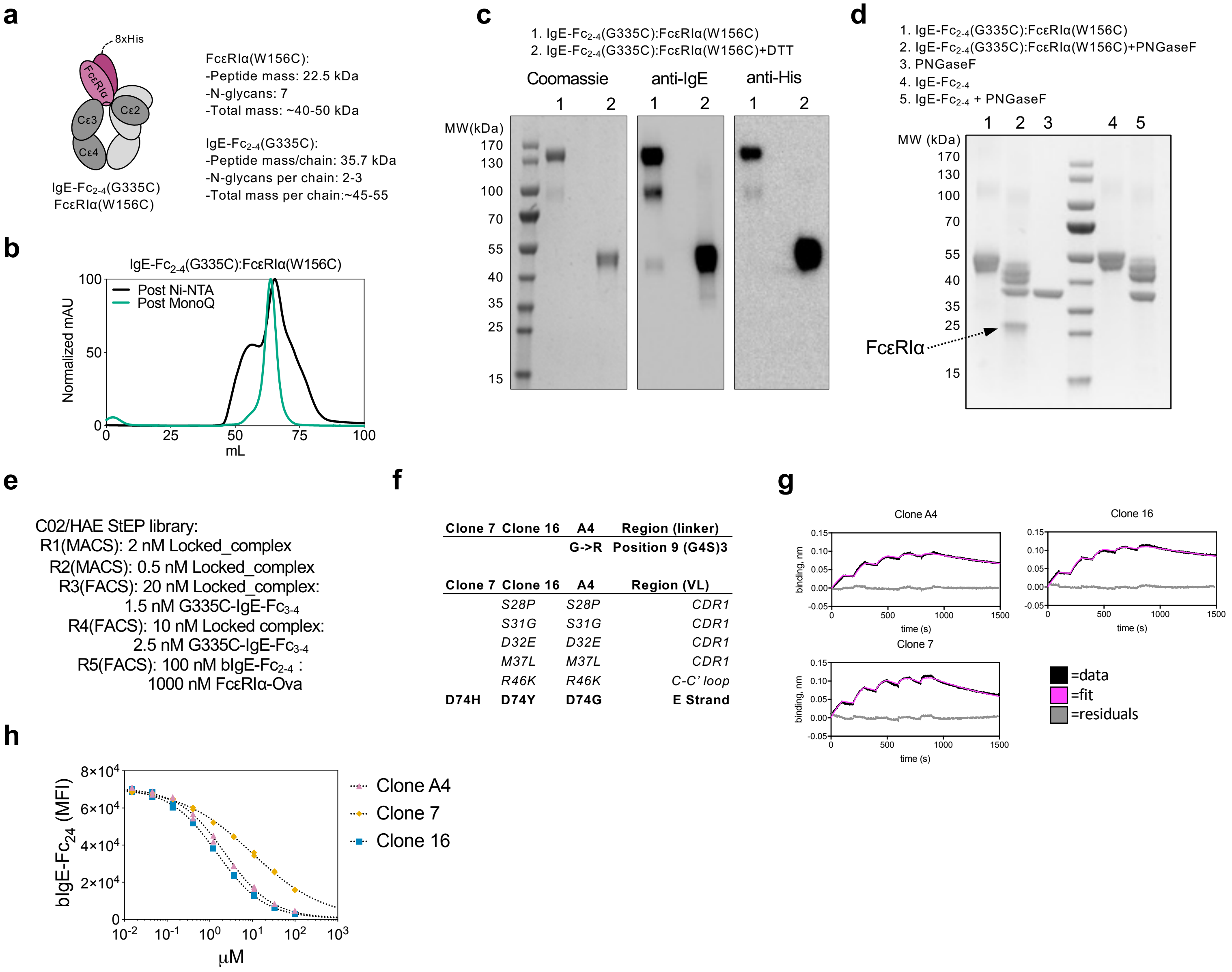

Supplementary Figure 5. Design and purification of IgE-Fc$_{2\text{-}4}$(G335C):FcεRIα(W156C) locked complex and selection of high affinity anti-locked complex variants. a Cartoon schematic of locked-complex with 8xHis tagged-FcεRIα(W156C) and un-tagged IgE-Fc$_{2\text{-}4}$ (G335C). Calculated peptide mass, number of known N-linked glycans, and total masses of human proteins. **b** SEC traces of products post Ni-NTA purification (black) and post MonoQ anion exchange (green). **c** Non-reducing and reducing SDS-PAGE (left), anti-IgE western blot (middle), and anti-His western blot (right) of locked complex. **d** Denaturing PNGaseF de-glycosylation of reduced locked complex or IgE-Fc$_{2\text{-}4}$. **e** Schematic of selection rounds conducted on C02/HAE StEP library. **f** Mutations in clones selected from the C02/HAE StEP library relative to C02 sequence. Mutations from shuffled HAE light chain are shown in italics, sporadic mutations are shown in bold. **g** Biotinylated IgE was immobilized on SA-tips and single cycle kinetic analysis (SCK) binding experiments were conducted on the Octet Red 96, exported, and fit in BiaEvaluation 3.0. Each SCK experiment contained five 100s association cycles, four 100s dissociation cycles, and a final 600s dissociation cycle. Experiments were conducted with two-fold serial dilutions from 50 nM to 1.56 nM, binding data was reference to blank tips exposed to analyte dilutions. **h** Disruption curves showing loss of bIgE-Fc$_{2\text{-}4}$ bound to FcεRIα-Ova coated polystyrene beads after 30-minute treatment with indicated variants. Curve fits (dotted line) from two replicate assays. Source data are provided as a Source Data file.

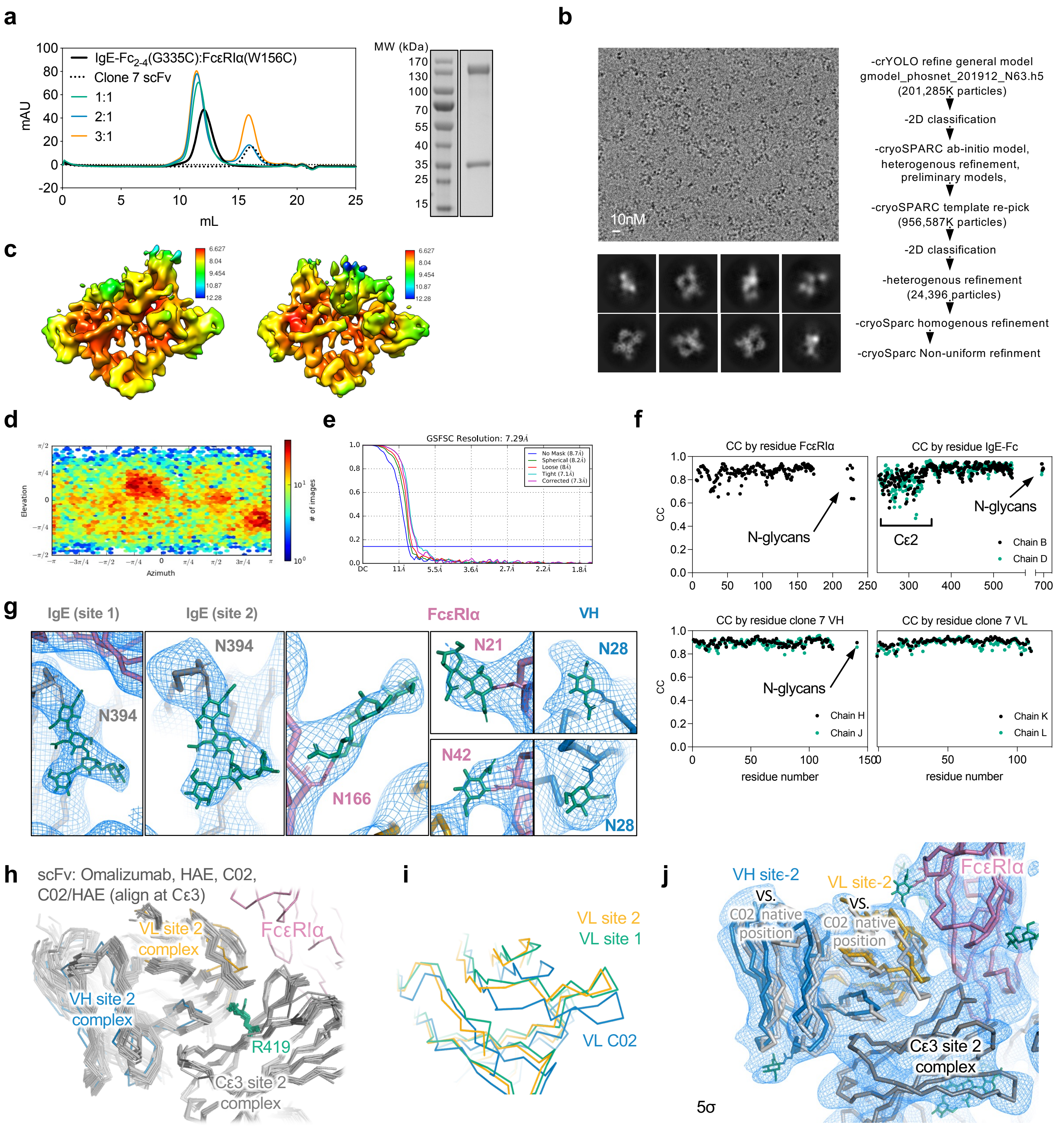

Supplementary Figure 6. Disruption-intermediate structural determination and analysis. **a** Clone 7 incubated with locked-complex in 0:1, 1:0, 1:1, 2:1, and 3:1 molar ratios and subject to SEC on Superdex S200 10/300 GL column. SDS-PAGE electrophoresis of ~10-12 mL peak from 2:1 complexes. **b** Representative micrograph 592 micrograph dataset, 2D averages from finally selected particles, and overview of data processing. **c** Local resolution estimates. **d** Angular distribution of particle projections from cryoSPARC. **e** Gold-standard FSC (cryoSPARC) with blue line at 0.143 FSC cut-off. **f** CC by residue for all chains in complex. **g** N-linked glycans (green) on IgE (grey), FcεRIα (magenta), and VH (blue) with glycosylation site labeled. Map contoured at 5σ. **h** All copies of the scFv:IgE interface in this publication were aligned to the site-1 proximal Cε3 domain of the disruption-intermediate model to assess the relative degree of VH and VL displacement from free-IgE structures. **i** The relative positions of the VL domain from both scFvs in the disruption-intermediate structure is displaced as compared the C02:IgE-Fc_{3-4} complex. **j** Cryo-EM density map contoured at 5σ and displacement of the clone 7 binding pose (blue: VH, yellow:VL) from the orientation observed in C02:IgE-Fc_{3-4} complex (grey).

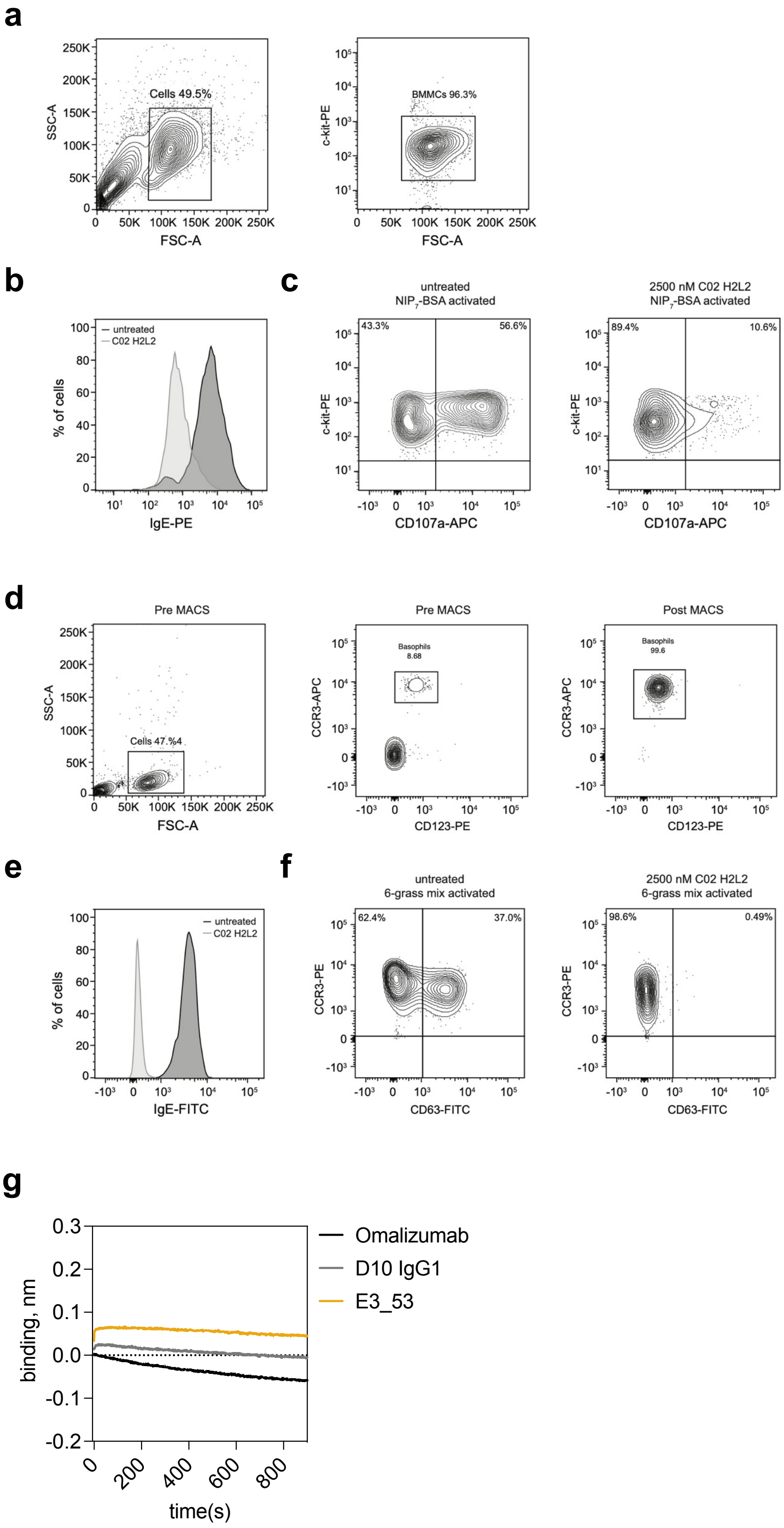

Supplementary Figure 7. Gating strategies for BMMC and basophil assays and controls for BLI disruption studies: a Flow cytometry analysis and gating strategy of BMMC. **b** Example flow plot of surface IgE removal from untreated and treated BMMC cultures. **c** Example flow plot of BMMC activation analysis (%CD107a+) in treated and untreated BMMC challenged with antigen. The same activation gating strategy was applied to anaphylactogenicity assays in which BMMC were treated with antibodies alone without antigen challenge (e.g., Fig. 7b). **d** Flow cytometry gating strategy and analysis of magnetically activated cell sorting (MACS) enrichment of primary human basophils. **e** Example flow plot of surface IgE removal from untreated and treated basophil cultures. **f** Example flow plot of basophil activation analysis (%CD63+) in treated and untreated basophils challenged with antigen. The same activation gating strategy was applied to anaphylactogenicity assays in which basophils were treated with antibodies alone without antigen challenge (e.g., Fig. 7c). **g** BLI studies of control non-competitive DARPin (E3_53), an off-target IgG1 antibody (D10 IgG1), and omalizumab binding to and/or disrupting immobilized IgE:FcεRIα complexes.

Supplementary Table 1: Tabulated kinetic data for IgE binding studies, Figure S1e

	K_D (M)	k_a($M^{-1}s^{-1}$)	k_d(s^{-1})	Rmax (nm)	Chi^2 (nm^2)
**Omalizumab Fab**	4.96E-09	9.13E+05	4.53E-03	0.142	2.26E-05
**Omalizumab scFv**	2.02E-09	9.44E+05	1.91E-03	0.0852	3.36E-05
**Omalizumab $scFv_{cc}$**	5.75E-09	1.21E+06	6.95E-03	0.0665	5.35E-06
**Omalizumab Fab_{H2L2}**	2.88E-09	1.14E+06	3.27E-03	0.112	2.01E-05
**E2_79**	1.18E-09	1.79E+06	2.12E-03	0.0482	1.41E-05

Supplementary Table 2: Tabulated curve fitting data for bead-based IgE disruption studies, Figure 1f,i and S1d

	ID_{50} (µM)	95% CI	R^2	ID_{50}/KD	95% CI
**Omalizumab IgG**	56.87	38.35-84.35	0.9227	*	*
**Omalizumab Fab**	47.15	34-63.53	0.9909	9506	6855-12808
**Omalizumab scFv**	21.02	15.92-25.91	0.9774	10406	7881-12827
**E2_79**	2.134	1.606-2.587	0.9774	1808	1361-2192
**E3_53**	1745	1085-2924	0.9763	*	*
**D10 IgG (control)**	interrupted			*	*
**Omalizumab $scFv_{cc}$**	39.26	29.67-49.54	0.9855	6828	5160-8616
**Omalizumab Fab_{H2L2}**	24.68	18.71-30.55	0.9922	8569	6496-10608

*Affinities not measured in this manuscript.

Supplementary Table 3: Tabulated kinetic data for IgE binding studies, Figure S3c

	K_D (M)	k_a($M^{-1}s^{-1}$)	k_d(s^{-1})	Rmax (nm)	Chi^2 (nm^2)
**813**	1.86E-09	7.97E+05	1.48E-03	0.079	3.47E-06
**C02**	9.50E-10	1.55E+06	1.48E-03	0.0774	4.44E-06
**HAE**	1.66E-10	1.52E+06	2.53E-04	0.0731	5.16E-06

Supplementary Table 4: Tabulated curve fitting data for bead-based IgE disruption studies, Figure S3d

	ID_{50} (µM)	95% CI	R^2	ID50/KD	95% CI
**813**	13.24	10.16-16.03	0.9912	7118	5462-8618
**C02**	2.483	1.925-2.959	0.9978	2614	2026-3115
**HAE**	1.361	1.068-1.609	0.9873	8199	6433-9693

Supplementary Table 5: Data collection and refinement statistics crystallography (molecular replacement)

	omalizumab scFv:IgE-Fc_{3-4} (7SHY)	clone 813 scFv:IgE-Fc_{3-4} (7SI0)	clone C02 scFv:IgE-Fc_{3-4} (7SHU)	clone HAE scFv:IgE-Fc_{3-4} (7SHZ)
**Data collection**				
Space group	C 1 2 1	P 21 21 21	C 2 2 21	I 1 2 1
Cell dimensions				
a, *b*, *c* (Å)	157.5 179.09 91.52	98.958 132.005 177.434	90.07 176.12 142.76	93.34 187.34 148.834
α, β, γ (°)	90 111.664 90	90 90 90	90 90 90	90 103.131 90
Resolution (Å)	39.93 - 3.0 (3.107 - 3.0)	39.59 - 3.0 (3.107 - 3.0)	49.19 - 2.75 (2.848 - 2.75)	47.35 - 3.0 (3.107 - 3.0)
R_{sym}	0.2864 (2.275)	0.145 (1.128)	0.09913 (1.423)	0.07024 (0.7036)
$CC_{1/2}$	0.989 (0.117)	0.996 (0.671)	0.999 (0.676)	0.998 (0.844)
I / s*I*	4.95 (0.14)	11.40 (1.61)	19.21 (1.83)	13.62 (1.94)
Completeness (%)	97.76 (98.20)	99.56 (99.87)	99.85 (99.73)	98.00 (99.00)
Redundancy	6.6 (6.8)	6.8 (7.1)	8.3 (8.4)	3.4 (3.6)
**Refinement**				
Resolution (Å)	39.93 - 3.0 (3.107 - 3.0)	39.59 - 3.0 (3.107 - 3.0)	49.18 - 2.75 (2.848 - 2.75)	47.35 - 3.0 (3.07 - 3.0)
No. reflections	46044 (4594)	47037 (4623)	29853 (2925)	48862 (4858)
R_{work} / R_{free} %	16.95 (36.40)/ 22.79 (45.85)	21.73 (33.84)/26.56 (37.77)	22.73 (32.13)/28.10 (33.99)	18.61 (39.48)/23.55 (40.73)
No. atoms				
Protein	13786	13773	6427	13741
Ligand/ion	343	258	115	320
Water	6	12	13	27
B-factors				
Protein	98.23	64.76	98.54	111.48
Ligand/ion	139.12	82.74	125.1	157.66
Water	67.97	37.06	67.4	82.43
R.m.s. deviations				
Bond lengths (Å)	0.011	0.006	0.003	0.007
Bond angles (°)	1.44	0.73	0.57	0.94

Data collected from a single crystal at 100˚K.

*Values in parentheses are for highest-resolution shell

Supplementary Table 6: Tabulated kinetic data for locked-complex binding studies, Figure 5g
*Limit hit fitting kd1, unable to calculate KD1

	K_{D1} (M)	$k_{a1}(M^{-1}s^{-1})$	$k_{d1}(s^{-1})$	k_{D2} (M)	$k_{a2}(M^{-1}s^{-1})$	$k_{d2}(s^{-1})$
**C02**	5.69E-08	9.58E+03	5.45E-04	1.13E-07	1.08E+05	1.22E-02
**HAE**	2.86E-09	9.63E+03	2.76E-05	2.58E-08	1.10E+05	2.83E-03
**Clone A4**	*	6.23E+03	0.00*	7.56E-08	2.95E+04	2.24E-03
**Clone 7**	*	1.34E+04	0.00*	2.26E-08	1.25E+05	2.82E-03
**Clone 16**	6.35E-10	9.26E+03	5.88E-06	1.51E-08	1.38E+05	2.09E-03

Supplementary Table 7: Tabulated curve fitting data for bead-based IgE disruption studies, Figure S5i

	ID_{50} (μM)	95% CI	R^2	ID50/KD	95% CI
**Clone A4**	2.544	1.935-3.053	0.996	6748	5133-8098
**Clone 7**	11.33	8.031-14.52	0.9968	19501	13823-24991
**Clone 16**	1.657	1.26-1.989	0.9976	5380	4091-6458

Supplementary Table 8: Tabulated kinetic data for IgE binding studies, Figure S5h

	K_D (M)	$k_a(M^{-1}s^{-1})$	$k_d(s^{-1})$	Rmax (nm)	Chi^2 (nm^2)
**Clone A4**	3.77E-10	1.50E+06	5.65E-04	0.0941	5.14E-06
**Clone 7**	5.81E-10	1.79E+06	1.04E-03	0.11	6.59E-06
**Clone 16**	3.08E-10	1.45E+06	4.48E-04	0.11	8.84E-06

Supplementary Table 9: Cryo-EM data collection, refinement and validation statistics

	Clone 7_2:FcεRIα:IgE-Fc2-4 (7SHT)
**Data collection and processing**	
Magnification	29,000
Voltage (kV)	300
Electron exposure (e–/Å^2)	44
Defocus range (μm)	-1.2 – -2.2
Pixel size (Å)	0.8521
Symmetry imposed	C1
Final particle images (no.)	24,396
Map resolution (Å)	7.29
FSC threshold= 1.43	
Map resolution range (Å)	12.28-6.627
**Refinement**	
Initial model used (PDB code)	1f6a (IgE Cε3, Cε4 and FcεRIα), 4j4p (Cε2), C02:IgE complex, this study, (clone 7 scFV)
Model resolution (Å)<br>FSC threshold=0.5	8.2
Model composition	
Non-hydrogen atoms	10078
Protein residues	1258
Ligands	20
B factors (Å^2)	
Protein	255.03
Ligand	375.03
R.m.s. deviations	
Bond lengths (Å)	0.007
Bond angles (°)	1.255
Validation	
MolProbity score	2.31
Clashscore	19.13
Poor rotamers (%)	0.64
Ramachandran plot	
Favored (%)	90.47
Allowed (%)	9.29
Disallowed (%)	0.24

Supplementary Table 10: Conformational rearrangements and displacements of Cε2 domains*

Structure	Rotation relative to IgE-Fc Cε2 dimer (1OOV) (°)	Displacement relative to IgE-Fc Cε2 dimer (1OOV) (Å)	Notes
**1OOV**	-	-	IgE-Fc
**5MOL**	1.17	0.72	IgE-Fc
**2WQR**	1.97	0.73	IgE-Fc
**2Y7Q**	3.53	1.28	IgE-Fc:FcεRIα complex
**5LGK**	24.58	4.85	IgE-Fc:CD23 complex
**5G64**	55.08	28.73	IgE-Fc:omalizumab complex
**7SHT clone_7:IgE:FcεRI**	69.41	35.49	IgE-Fc:clone7:FcεRIα complex
**4J4P**	123.92	51.72	IgE-Fc:aεFab complex
**6EYO**	130.06	47.14	8D6:IgE-Fc complex

*Calculated following alignment to Cε2 proximal Cε3 domain and quantified using angle between domain script from PSICO to compare Cε2 dimer displacements

Supplementary Table 11: Tabulated curve fitting data for DARPin disruptive inhibitors in bead-based disruption assay, adapted from "Structure-guided design of ultrapotent disruptive IgE inhibitors to rapidly terminate acute allergic reactions." *J. Allergy Clin. Immunol.* 148, (2021). Included for comparison to antibody variants presented in bead-based disruption assays in table S2, S4, and S7.

	ID_{50} (μM)*	95% CI	R^2
**E2_79**	2.010	1.763-2.293	0.9931
**E3_53**	36155	2.206-undefined	0.7808
**E2_79+E3_53**	0.872	0.767 - 0.990	0.9963
**bi53_79**	0.086	0.074 - 0.010	0.9941
**biE07_79**	0.015	0.012 - 0.018	0.9955
**tri11_53_79**	0.300	0.245 - 0.374	0.9825
**tri11_E07_79**	0.079	0.067 - 0.093	0.9896
**KiH_E07_79**	0.012	0.011 - 0.014	0.9974

*Fit values derived from Figures 1H and 3B from Structure-guided design of ultrapotent disruptive IgE inhibitors to rapidly terminate acute allergic reactions. J. Allergy Clin. Immunol. 148, (2021).

Supplemental gel and blot source data: Uncropped blots for cropped figures S5c and S6a

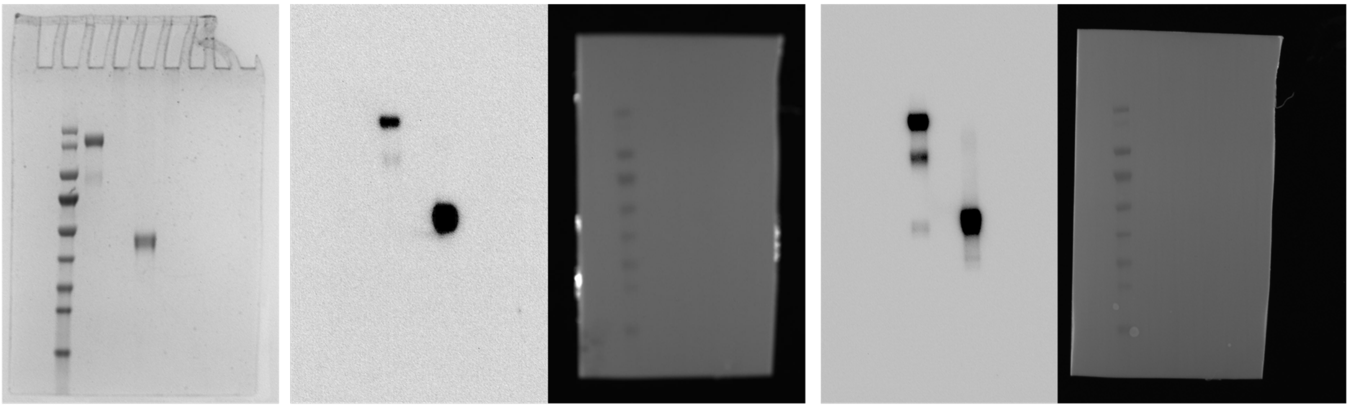

For Supplementary Figure 5c. Gels and blots: Coomassie, anti-IgE and associated brightfield image for ladder, anti-His and associated brightfield image.

Lane 1: MW marker
Lane 2 Complex of IgE-Fc2-4(G335C):FcεRIα(W156C)
Lane 3 Complex of IgE-Fc2-4(G335C):FcεRIα(W156C), reduced

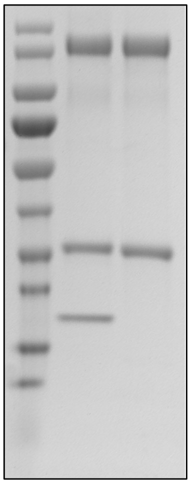

For Supplementary Figure 6a. Gel.
Lane 1: MW marker
Lane 2: Complex of IgE-Fc2-4(G335C):FcεRIα(W156C), clone 7 scFv, E07 anti-IgE DARPin (not used in publication)
Lane 3: complex of IgE-Fc2-4(G335C):FcεRIα(W156C) , clone 7 scFv
