# Supplementary material for: Directed evolution of and structural insights into antibody-mediated disruption of a stable receptor-ligand complex
Source: Nat Commun. 2021 Dec 3;12:7069. doi: 10.1038/s41467-021-27397-z (PMC8642555; doi:10.1038/s41467-021-27397-z)
Supplement: Supplementary file 3 — Reporting Summary [file 41467_2021_27397_MOESM3_ESM.pdf]

nature research

Corresponding author(s): *Double-blind peer review submissions: write DBPR and your manuscript number here instead of author names.*

Last updated by author(s): *YYYY-MM-DD*

Reporting Summary

Nature Research wishes to improve the reproducibility of the work that we publish. This form provides structure for consistency and transparency in reporting. For further information on Nature Research policies, see our Editorial Policies and the Editorial Policy Checklist.

Statistics

For all statistical analyses, confirm that the following items are present in the figure legend, table legend, main text, or Methods section.

n/a	Confirmed	
☐	☒	The exact sample size (*n*) for each experimental group/condition, given as a discrete number and unit of measurement
☐	☒	A statement on whether measurements were taken from distinct samples or whether the same sample was measured repeatedly
☐	☒	The statistical test(s) used AND whether they are one- or two-sided<br>*Only common tests should be described solely by name; describe more complex techniques in the Methods section.*
☒	☐	A description of all covariates tested
☐	☒	A description of any assumptions or corrections, such as tests of normality and adjustment for multiple comparisons
☐	☒	A full description of the statistical parameters including central tendency (e.g. means) or other basic estimates (e.g. regression coefficient) AND variation (e.g. standard deviation) or associated estimates of uncertainty (e.g. confidence intervals)
☐	☒	For null hypothesis testing, the test statistic (e.g. *F*, *t*, *r*) with confidence intervals, effect sizes, degrees of freedom and *P* value noted<br>*Give P values as exact values whenever suitable.*
☒	☐	For Bayesian analysis, information on the choice of priors and Markov chain Monte Carlo settings
☒	☐	For hierarchical and complex designs, identification of the appropriate level for tests and full reporting of outcomes
☒	☐	Estimates of effect sizes (e.g. Cohen's *d*, Pearson's *r*), indicating how they were calculated

Our web collection on statistics for biologists contains articles on many of the points above.

Software and code

Policy information about availability of computer code

Data collection	EM data collection was performed using Serial EM v. 3-6. Crystallography data collection was performed using Blue Ice v. 5. Other data collection was performed using Octet Data Aquisition v. 7.1, BD FACSDiva v8.0, and GraphPad Prism v. 8-9.
Data analysis	Data analysis was performed using MotionCor2, crYOLO, CyroSparc v. 3.0, Coot v. 0.8, and Phenix v. 1.14, Microsoft Excel v. 14-16, GraphPad Software Prism v. 8-9, PyMol v. 2.4, UCSF Chimera v. 1.15, Octet Data Analysis v. 7.1, BIAevaluation, Clamp XP, and FlowJo v 10.3.

For manuscripts utilizing custom algorithms or software that are central to the research but not yet described in published literature, software must be made available to editors and reviewers. We strongly encourage code deposition in a community repository (e.g. GitHub). See the Nature Research guidelines for submitting code & software for further information.

Data

Policy information about availability of data

All manuscripts must include a data availability statement. This statement should provide the following information, where applicable:

- Accession codes, unique identifiers, or web links for publicly available datasets
- A list of figures that have associated raw data
- A description of any restrictions on data availability

There are no restrictions on data availability. All structures and models from this work have been deposited at the PDB (link: https://www.rcsb.org) under the following accession numbers: 7SHY, 7SI0, 7SHU, 7SHZ, 7SHT. Datasets from the PDB used in this work include 1F6A, 4GRG, 6NMS, 6NMU, 3B2U, 3C09, 3POY, 7MXI, 2Y7Q, 5G64, 10OV, 5MOL, 2WQR, 5LGK, 4J4P, 6EYO.

All main figures are derived from underlying raw data with the exception of representative flow cytometry gating plots and when possible raw data is supplied in source data file.

Field-specific reporting

Please select the one below that is the best fit for your research. If you are not sure, read the appropriate sections before making your selection.

☒ Life sciences ☐ Behavioural & social sciences ☐ Ecological, evolutionary & environmental sciences

For a reference copy of the document with all sections, see nature.com/documents/nr-reporting-summary-flat.pdf

Life sciences study design

All studies must disclose on these points even when the disclosure is negative.

Sample size	No power calculations were performed. Sample size was determined based on previous experiments and expected significance levels.
Data exclusions	One data point was excluded (see source data figure f), otherwise all data included unless sample was lost (as indicated).
Replication	Individual experiments were repeated at least twice with similar results, unless otherwise stated. Data is shown as representation of one experiment or as a combination of multiple independent experiments, as indicated in the text.
Randomization	Samples were chosen and assigned in an unbiased and randomized way.
Blinding	The investigators were not blinded during experimentation because of lack of personnel involved directly in the project.

Reporting for specific materials, systems and methods

We require information from authors about some types of materials, experimental systems and methods used in many studies. Here, indicate whether each material, system or method listed is relevant to your study. If you are not sure if a list item applies to your research, read the appropriate section before selecting a response.

Materials & experimental systems

n/a	Involved in the study	
☐	☒	Antibodies
☐	☒	Eukaryotic cell lines
☒	☐	Palaeontology and archaeology
☒	☐	Animals and other organisms
☐	☒	Human research participants
☒	☐	Clinical data
☒	☐	Dual use research of concern

Methods

n/a	Involved in the study	
☒	☐	ChIP-seq
☐	☒	Flow cytometry
☒	☐	MRI-based neuroimaging

Antibodies

Antibodies used

anti-CD117-PE at 1:2000 dilution (clone 2B8, Thermo Fisher Scientific, MA, USA, Cat# 12-1171-82)
anti-human FcεRIα-APC at 1:400 dilution (antibody clone AER-37, Thermo Fisher Scientific, MA, USA, Cat# 17-5899-42)
anti-mouse CD107a-APC at 1:300 dilution (clone 1D4B, BioLegend, San Diego, California, USA Cat# 121614)
anti-human CD193-PE and anti-human CD63-FITC antibodies from Flow CAST® kit at 1:10 dilution (Bühlmann Laboratories AG, Cat# FK-CCR)
anti-CD123-PE at 1:800 dilution (clone 9F5, BD Biosciences Pharmingen, San Diego, CA, USA, Cat# 555644)
anti-CD193-APC at 1:400 dilution (clone 5E8, BioLegend, San Diego, California, USA, Cat# 310708)
anti-mouse Ig light chain-PE (clone RML-42, BioLegend, San Diego, California, USA, Cat# 407308)
anti-human IgE-PE at 1:400 dilution (Thermo Fisher Scientific, MA, USA, cat# 12-6986-42)

Recombinant antibodies: Human chimeric NIP-specific IgE at variable dilutions (clone JW8, NBS-C BioScience, Vienna, AUT)

For yeast studies secondary antibodies were titrated to achieve maximum separation of positive and negative populations.
Streptavidin AF-647 conjugate at 1:400 dilution (ThermoFisher, MA, USA, Cat#S21374)
anti-Ova FITC conjugate 1:50 dilution (Rockland, PA, USA, cat# 200-4233-0100)
anti-cMyc at 1:400 dilution (Gallus Immunotech, MA, USA cat# ACMYC)
anti-chicken PE 1:200 dilution (EMD Millipore, MA, USA, cat# AP503H).

Validation

anti-CD117-PE at 1:2000 dilution (clone 2B8, Thermo Fisher Scientific, MA, USA, Cat# 12-1171-82)
Validation: "Each lot of this antibody is quality control tested by immunofluorescent staining with flow cytometric analysis"
anti-human FcεRIα-APC at 1:400 dilution (antibody clone AER-37, Thermo Fisher Scientific, MA, USA, Cat# 17-5899-42)

Validation: None listed, flow data staining human blood vs isotype controls presented at manufactured website.
anti-mouse CD107a-APC at 1:300 dilution (clone 1D4B, BioLegend, San Diego, California, USA Cat# 121614)
Validation: "Each lot of this antibody is quality control tested by immunofluorescent staining with flow cytometric analysis."" This monoclonal antibody was raised against NIH/3T3 mouse embryonic fibroblast tissue culture cell membranes. It has been mapped to the N-terminus of LAMP-1."
anti-human CD193-PE and anti-human CD63-FITC antibodies from Flow CAST® kit at 1:10 dilution (Bühlmann Laboratories AG, Cat# FK-CCR)
"Validation: Validation for individual reagents not listed. For kit "Precision (Sample Background) 16.2 %CV. One sample incubated 20 times and subsequently analyzed. Mean: 2.4%; SD: 0.4% . Precision (Positive Control) 5.4 %CV One sample incubated 20 times and subsequently analy- sed. Mean: 35.5%; SD: 1.9% . Inter Technician Variation 3.7-8.1%CV Two samples tested by five technicians within the same day: Mean: 69.6%; SD: 2.6% Mean: 48.1%; SD: 3.9%"
anti-CD123-PE at 1:800 dilution (clone 9F5, BD Biosciences Pharmingen, San Diego, CA, USA, Cat# 555644)
Validation "QC Testing: Human." "Application: flow cytometry, routinely tested."
anti-CD193-APC at 1:400 dilution (clone 5E8, BioLegend, San Diego, California, USA, Cat# 310708)
Validation: "Each lot of this antibody is quality control tested by immunofluorescent staining with flow cytometric analysis."
anti-mouse Ig light chain-PE (clone RML-42, BioLegend, San Diego, California, USA, Cat# 407308)
Validation: "Each lot of this antibody is quality control tested by immunofluorescent staining with flow cytometric analysis." "The RML-42 monoclonal antibody reacts with immunoglobulin light chain lambda in all tested mouse haplotype (Igh-a and b). It does not react with kappa chain."
anti-human IgE-PE at 1:400 dilution (Thermo Fisher Scientific, MA, USA, cat# 12-6986-42)
Validation: "This Ige21 antibody has been pre-titrated and tested by flow cytometric analysis of human peripheral blood cells."

Recombinant antibodies: Human chimeric NIP-specific IgE at variable dilutions (clone JW8, NBS-C BioScience, Vienna, AUT)
Validation: Not listed, recombinant source well established reagent.

For yeast studies secondary antibodies were titrated to achieve maximum separation of positive and negative populations.
Streptavidin AF-647 conjugate at 1:400 dilution (ThermoFisher, MA, USA, Cat#S21374)
Validation: None listed, flow data staining presented at manufactured website.
anti-Ova FITC conjugate 1:50 dilution (Rockland, PA, USA, cat# 200-4233-0100)
Validation: "Ovalbumin Antibody is an IgG fraction antibody purified from monospecific antiserum by a multi-step process which includes delipidation, salt fractionation and ion exchange chromatography followed by extensive dialysis against the buffer stated above. Assay by immunoelectrophoresis resulted in a single precipitin arc against anti-fluorescein, anti-Rabbit Serum as well as purified and partially purified Ovalbumin [Hen Egg White]. Cross reactivity against Ovalbumin from other tissues and species may occur but have not been specifically determined."
anti-cMyc at 1:400 dilution (Gallus Immunotech, MA, USA cat# ACMYC)
Validation: "Chickens were immunized with EQKLISEEDL (C-MYC) conjugated to KLH. After multiple immunizations in Freunds adjuvant, eggs were collected and the immunoglobulin fraction prepared. Antibodies were immunoaffinity purified using the peptide immobilized on a solid support."
anti-chicken PE 1:200 dilution (EMD Millipore, MA, USA, cat# AP503H).
Validation: "Purified by affinity chromatography on chicken IgG covalently linked to agarose." "The antibody reacts with the heavy and light chains of chicken IgG as demonstrated by ELISA."

Eukaryotic cell lines

Policy information about cell lines

Cell line source(s)	293-6E cells, National Research Council Canada and Sf9 cells, ThermoFisher
Authentication	Cell lines were obtained from the NRC Canada and used without further verification.
Mycoplasma contamination	293-6E cells have been tested for mycoplasma contamination by PCR in ISO/IEC 17025 certified laboratory at ATCC.
Commonly misidentified lines (See ICLAC register)	HEK-6E cells (derived from HEK cells), however these were only used for protein expression and were obtained from NRC

Human research participants

Policy information about studies involving human research participants

Population characteristics	Whole blood donations from allergic volunteers were collected. All seven donors included in this study were >18 years of age and had a proven record of grass allergy. Neither gender nor age of the donors affects basophil reactivity and thus these parameters can be excluded as covariate-relevant characteristics.
Recruitment	Patients were randomly recruited through public advertisement at the University Hospital Bern, Switzerland. The donors had a proven record of grass allergy and their basophils were known to be activated upon allergen provocation. Choosing donors with known grass allergies was critical for this study, since the goal has been to assess the inhibitory effect of different disruptive anti-IgE antibodies on allergen mediated basophil activation.
Ethics oversight	The study was approved by "swissethics" (the umbrella organisation of the Cantonal Ethics Committees) and the Local Ethics Committee of the Canton Bern (license: KEK 2018-00204).

Note that full information on the approval of the study protocol must also be provided in the manuscript.

Flow Cytometry

Plots

Confirm that:

- [x] The axis labels state the marker and fluorochrome used (e.g. CD4-FITC).
- [x] The axis scales are clearly visible. Include numbers along axes only for bottom left plot of group (a 'group' is an analysis of identical markers).
- [x] All plots are contour plots with outliers or pseudocolor plots.
- [x] A numerical value for number of cells or percentage (with statistics) is provided.

Methodology

Sample preparation

Human basophil isolation: Human peripheral whole blood was collected from volunteers with grass allergies who received informed consent in accordance with the Helsinki Declaration, and the study was approved by the local ethics committee. Primary human basophils were isolated from whole blood using Percoll density centrifugation of dextran-sedimented supernatants. Subsequently, the basophils were further enriched with negative selection using the Basophilic Isolation Kit II from Milteny (Miltenyi Biotec, Bergisch Gladbach, Germany). As human basophil culture medium (RPMI+/+) RPMI 1640 medium (Biochrome) was used, supplemented with 10 % Hyclone FCS (Fisher Scientific), penicillin 100 U per mL, 100 µg per mL streptomycin and 10mM HEPES buffer (Stock Solution 1M, Life Technologies) and recombinant human IL 3 from Peprotec (United States). The study was approved by the local ethics committee (KEK 2018-00204).

Bone marrow-derived mast cell (BMMC) cultures: Culture, characterization and differentiation of transgenic genes hFcεRIα+ BMMCs (BMMCtg) from huFcεRIα-transgenic mice ((B6.Cg-Fcer1atm1Knt Tg(FCER1A)1Bhk/J) were derived by 3 to 4 weeks cultivation of bone marrow cells of the femur in BMMC culture medium (MC/9 media supplemented with 1mM sodium pyruvate, 200mM L-glutamine, 1x non-essential amino acids, 50µM β-Mercaptoethanol, 30ng/ml recombinant IL-3 of the mouse). BMMCs were characterized by flow cytometry as CD117+ (antibody clone 2B8, Thermo Fisher Scientific, MA, USA), human FcεRIα+ cells (antibody clone AER-37, Thermo Fisher Scientific, MA, USA).

Disruptive potency and anaphylactogenicity assay with BMMCstg : First, the BMMCstg were sensitized with 3nM human NIP-specific IgE-JW8 (murine lambda chain) overnight at 37° C in a 5% CO2 atmosphere. Following sensitization, the cells were washed three times with phosphate buffer saline (PBS) pH 7.4. Subsequently, disruptive anti-IgE inhibitors (156nM-2500nM) were diluted in supplemented BMMC culture medium MC/9 and added to the sensitized cells for 6 hours at 37°C in 5% CO2 atmosphere. The cells were then washed three times with PBS and divided into two fractions. The first fraction was used to measure the remaining surface IgE with a monoclonal anti-mouse anti-murine lambda chain - PE and the anaphylactogenicity by an activation marker staining. The second fraction of treated BMMCstg were stimulated with 100ng/mL NIP(7)-BSA (Biosearch Technologies, INC) in the presence of an activation marker stain.

Anaphylactogenicity, disruptive efficacy and inhibitory potency with BMMCtg: To evaluate the anaphylactogenicity and the disruptive potency of the disruptive anti-IgE-DARPins we cultured isolated primary human basophils at a density of 1x106 cells/ml per well in a round bottom plate with 96 wells in RPMI+/+ supplemented with 10 ng/ml recombinant human IL-3 (rhuIL-3) overnight at 37°C. To assess the influence of IgE disruption on allergen induced basophil activation, we first defined the 6-grass allergen mix concentration (Bühlmann Laboratories AG, Schönenbuch, CH) at which suboptimal activation (ECSubopt.) can be achieved for each individual donor. For this purpose, we cultivated 25'000 basophils in RPMI+/+ medium, which was composed with the 6-grass allergen mix, IL-3, and the Flow CAST® Kit antibody stain (CD63 and CCR3), for 30min at 37°. Subsequently, we measured activated cells (CD63+ cells) by flow cytometry. In the next step, 50,000 basophils were pre-incubated for 6 hours with the disruptive anti-IgE inhibitors at 37°C in RPMI+/+ medium with rhuIL-3. Upon incubation, the cells were washed three times with PBS and divided into two fractions. The first fraction was used to measure the remaining surface IgE with an anti-human IgE-PE antibody (clone Ige21, Thermo Fisher Scientific, MA, USA) and for anaphylactogenicity with an anti-human LAMP3 FITC (CD63). We used the second fraction to stimulate the cells with the predetermined ECSubopt of the 6-grass allergen mixture in the presence of a Flow CAST® kit antibody stain mix (activation staining of CD63). Finally, we determined activated basophil frequencies and surface measurements by flow cytometry.

Yeast were induced overnight and washed with PBS with 0.1% w/v BSA, 2.5mM EDTA, with penicillin 100 U/mL and Streptomycin 100 µg/mL prior to staining.

*In yeast experiments, often with multiple sample comparisons, contour plots were not employed and numerical values for gates are not employed. Most of these figures represent clonal populations of yeast and gates and population percentages are not used to make experimental conclusions.

Instrument	Flow cytometry on basophils and BMMC was performed using a BD FACSCanto device (BD Bioscience, Franklin Lakes, NJ, USA). For yeast all experiments were performed on Accuri C6 Cytometer.
Software	Results were evaluated with FlowJo Version 10.1 (Ashland, OR, USA)and BD FACSDIVA™ Software.
Cell population abundance	- basophils in whole blood <1%<br>- purified primary human basophils: > 90%<br>- differentiated BMMCs: >90%
Gating strategy	- purified primary human basophils: FSC(low), CD123+, CD193+ cells<br>- BMMCs: mouse c-kit+, human FceRIa+ cells<br>-Yeast experiments: yeast cells by FSC/SSC , singlets by SSC-A/SSC-H, and cMyc+ cells gaiting on non-induced cMyc- yeast.

☒ Tick this box to confirm that a figure exemplifying the gating strategy is provided in the Supplementary Information.
